# Correlation of FT-IR Fingerprint and α-Glucosidase Inhibitory Activity of Salak (*Salacca zalacca*) Fruit Extracts Utilizing Orthogonal Partial Least Square

**DOI:** 10.3390/molecules23061434

**Published:** 2018-06-13

**Authors:** Mohammed S. M. Saleh, Mohammad Jamshed Siddiqui, Siti Zaiton Mat So’ad, Fatimah Opeyemi Roheem, Salima Saidi-Besbes, Alfi Khatib

**Affiliations:** 1 Department of Pharmaceutical Chemistry, Kulliyyah of Pharmacy, International Islamic University Malaysia (IIUM), Indera Mahkota, Kuantan 25200, Pahang, Malaysia; ksm20085@hotmail.com (M.S.M.S.); dszaiton@iium.edu.my (S.Z.M.S.); bukolami_fatty@yahoo.com (F.O.R.); alfikhatib@iium.edu.my (A.K.); 2 Laboratoire de Synthèse Organique Appliquée, Faculté des Sciences Exactes et Appliquées, Département de Chimie, Université Oran1, BP 1524 El Mnaoueur, 31000 Oran, Algérie; salima_saidi@yahoo.fr

**Keywords:** α-glucosidase inhibitory activity, fingerprint, Fourier transform infrared spectroscopy, salak fruit

## Abstract

Salak fruit (*Salacca zalacca*), commonly known as snake fruit, is used indigenously as food and for medicinal applications in Southeast Asia. This study was conducted to evaluate the α-glucosidase inhibitory activity of salak fruit extracts in correlation to its Fourier transform infrared spectroscopy (FT-IR) fingerprint, utilizing orthogonal partial least square. This calibration model was applied to develop a rapid analytical method tool for quality control of this fruit. A total of 36 extracts prepared with different solvent ratios of ethanol–water (100, 80, 60, 40.20, 0% *v*/*v*) and their α-glucosidase inhibitory activities determined. The FT-IR spectra of ethanol–water extracts measured in the region of 400 and 4000 cm^−1^ at a resolution of 4 cm^−1^. Multivariate analysis with a combination of orthogonal partial least-squares (OPLS) algorithm was used to correlate the bioactivity of the samples with the FT-IR spectral data. The OPLS biplot model identified several functional groups (C–H, C=O, C–N, N–H, C–O, and C=C) which actively induced α-glucosidase inhibitory activity.

## 1. Introduction

Salak [*Salacca zalacca* (Gaertn.) Voss] is commonly known as snake fruit, due to its brown skin and widespread availability in Southeast Asian countries. However, it is also known with a variety of names in Asia, North and South America, and European countries, such as snake palm and salak palm (English); keshi sa laka and she pi guo zong (Chinese); fruit à peau de serpent, fruit de palmier à peau de serpent and salacca aux fruit à peau de serpent (French); salakpalme, salak, schlagenfrucht and zalak (German); sarakka yashi (Japanese); salaca (Spanish) and fruta cobra (Portuguese) [1]. This species of palm tree belongs to the family of Arecaceae. The fruit looks like an egg in shape, weighing around 30–100 g with a 3–4 cm diameter. This fruit is an essential source of food, proteins, fats, fibers, phenolic compounds, and carbohydrates [2]. One of the previous research studies reported that various classes of phytochemicals, like flavonoid, alkaloid, terpenoid, tannin, and quinone were present in the salak fruit [3]. Salak fruit is traditionally used by Malaysians to treat diabetes. Several studies reported that the salak fruit possesses anticancer, antihyperuricemic, antioxidant, antidiabetic, and proliferative activities [3,4,5,6,7]. Gorinstein and associates performed in vitro and in vivo studies where they compared the antioxidant properties of rare exotic Thai fruits with conventional fruits, and found that there was a significant reduction of plasma lipid profile, as well as maintained plasma antioxidant activity [6]. Zubaidah et al. reported that *Salacca* vinegar influences the lipid profile levels of diabetic rats which influence the decrease of LDL, triglycerides, total cholesterol, and increases the HDL [8]. Rohaeti and associates reported that the salak fruit has α-glucoside inhibitory activity [9], and therefore, could be a therapeutic agent in combatting hyperglycemia related problems.

The α-glucosidase is the most important enzyme involved in the process of digestion of carbohydrates into glucose. This enzyme breaks down disaccharides and complex carbohydrates through hydrolysis [10]. Intestines are the principal organs which produce the α-glucosidase enzyme, and this enzyme reacts with non-reducing sugars, and of its substrates into glucose monomers [11].

Different spectroscopic techniques, like nuclear magnetic resonance spectroscopy (NMR), gas chromatography-mass spectrometry (GC-MS), liquid chromatography-mass spectrometry (LC-MS) and high-performance liquid chromatography (HPLC), are generally used to perform metabolite profiling analyses [12,13,14,15].

A more useful alternative method of qualifying plant extracts or any products is Fourier transform infrared spectroscopy FT-IR. It is simple, rapid, and smooth in operation [16]. FT-IR is capable of identifying functional groups. Moreover, FT-IR has been proposed in ecotoxicology and environmental microbiology [17]. Another advantage is the use of less reagent, and it is entirely non-destructive. FT-IR is suitable to investigate samples with specificity and high reproducibility. It is a comparatively easier method and requires minimal sample preparation [17,18]. The theory is based on the absorption of radiation by the functional groups present in the analyte at a specific wavelength, and vibration may be in the form of bending or stretching, which leads to a spectrum or fingerprint profile [19].

A convenient statistical tool for the analysis of large datasets derived from spectroscopic tools is multivariate data analysis, or simply, MVDA. This tool is widely implemented in numerous pharmaceutical and chemical studies, including pharmaceutical chemistry, clinical metabolism, quality control [20,21], screening of biomarkers [22], environmental metabolism, toxicity [23], chemotaxonomy [24,25], and a study classifying samples by their phytochemical composition. Nowadays, it is possible to manage a significant dataset by employing projection-based MVDA.

The study aimed to generate a fingerprint profile using FT-IR spectroscopy combined with a multivariate predictive model based on orthogonal partial least square (OPLS) for rapid investigation of α-glucosidase inhibitory activities of the salak (*S. zalacca*) fruit, and also predict the IC_50_ value. The OPLS model executes correlation between the functional groups and α-glucosidase inhibitory activity.

## 2. Results and Discussion

### 2.1. Extraction Yield 

Salak fruit extracts were obtained from different solvent ratios of ethanol–water and shown in Table 1. The percentage yield of 0% ethanol–water and other extracts were significantly different from one another (*p* < 0.05). This clearly showed that the percentage yield of the extracts is totally dependent on the polarity of the extracting solvent. The extracts’ percentage yield ranged between 20–70%. The 80% solvent ratio of ethanol–water was observed to have the highest yield, while 100% ethanol extract has the lowest. This result indicated that a higher polarity is not necessarily a prerequisite for best extraction yield, but rather, the solvent of medium polarity. The result also infers that a high percentage of ethanol may be the most suitable solvent for maximum extraction. The percentage yield followed this trend 80% > 60% > 0% > 40% > 20% > 100%. The result was in agreement with [26,27].

### 2.2. α-Glucosidase Inhibitory Activity of the Salak Fruit Extracts 

α-Glucosidase inhibitory activity was reported as IC_50_ value (μg/mL), with the lowest value indicating the highest inhibition. The extracts of different ratios of ethanol–water were evaluated based on their ability to inhibit the α-glucosidase enzyme, as presented in Table 1. The IC_50_ values of salak fruit extracts ranged from 15.94 to 271.46 μg/mL. The inhibition potential of 100%, 80%, and 60% ethanol–water extracts showed no significant differences (*p* > 0.05). The α-glucosidase inhibitory activity results showed the extracts of 0%, 20%, and 40% ethanol–water showed significant differences (*p* < 0.05). The results indicated that as the ratio of water increased in the extract solvent, the α-glucosidase inhibitory activity decreased significantly. However, 60% ethanol–water extract exhibited the highest inhibition with IC_50_ value 15.94 ± 2.52 μg/mL, followed by 100% ethanol 19.15 ± 1.82 μg/mL, the extracts were not significantly different from the positive control (quercetin) (*p* < 0.05). The lowest α-glucosidase inhibitory activity observed in the 0% extract was 271.46 ± 15.55 μg/mL, due to the hydrophilic property of water. Inhibitory activity of all extracts was observed in the following trend 60% > 100% > 80% > 20% > 40% > 0%. This result may be due to the phytochemical constituents being responsible for the α-glucosidase inhibitory activity. 

### 2.3. Analysis of Infrared Spectra

**Figure 1:**
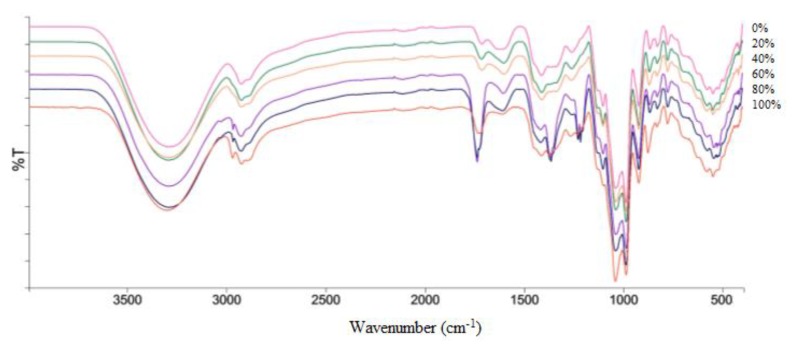
Representative infrared spectra of salak fruit extracts 0%, 20%, 40%, 60%, 80%, and 100% ethanol in water.

Infrared spectrometry is used to obtain the functional group characteristics and structural information of a metabolite present in an extract [28]. It is used as an analytical instrument for quality control purposes. FT-IR spectra of different extracts obtained from the salak fruit were presented in Figure 1. The range of the absorption band of each extract seems identical by mere observation, because of similarities in their chemical composition. However, there are obvious differences when studied critically. This infers that the absorption bands of the spectra about α-glucosidase inhibition may not be detected by mere observation evaluation. There are underlined differences in the peaks due to the presence of different functional groups. The O–H stretching vibrations in carboxylic acid, alcohol, and phenols groups produce a strong band in the 3220 to 3540 cm^−1^ region of the spectrum; compounds containing this functional group have been previously reported from this fruit (3-hydroxystigmastan-5(6)-en β-sitosterone gallic acid, epicatechin, caffeic acid, chlorogenic acid, linoleic acid) [3,29,30]. The frequency and intensity of aldehyde, alkanes, alkenes, alkyne, and aromatics stretching absorption in the region of 2928 to 2969 cm^−1^. Stretching absorption bands for the carbonyl groups (C=O) (β-sitosterone) [29], as carboxylic acids, aldehydes, esters, and ketones, appear in the region of 1620 to 1720 cm^−1^. Vibrations for the conjugation C=C stretch at 1555 cm^−1^. Medium absorption at 1500 cm^−1^ characterizes the peak of secondary amines (N–H) (levodopa,5-(methoxycarbonyl)-1H-pyrrole-3-carboxylic acid). Sulfones, sulfonamides, sulfonyl chlorides, and sulfates (S=O) stretch in the range of 1330–1140 cm^−1^. However, none of these groups have been reported to be from this fruit previously. Anhydrides, carboxylic, alcohols, ethers, esters display a strong bond (C–O) (ascorbic acid) [31] bending band of 1000 to 1152 cm^−1^. The peak from 700 to 920 cm^−1^ is due to the presence of alkanes and aromatics (C–H) out of the plane. Strong halogen groups (C–X) are at 500–700 cm^−1^ [32].

### 2.4. OPLS Modeling

**Figure 2:**
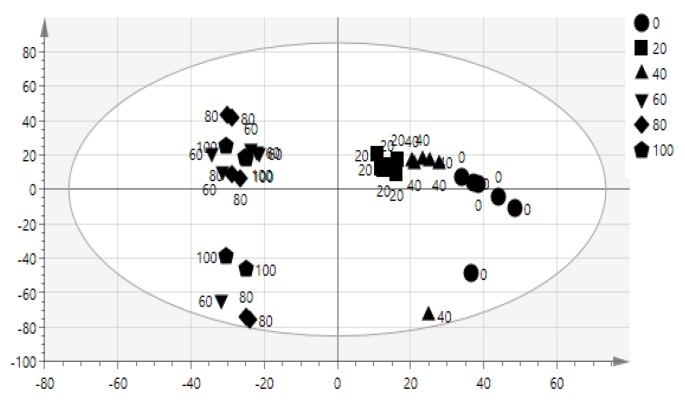
Orthogonal partial least-squares (OPLS) score plot of different concentration of salak extracts.

**Figure 3:**
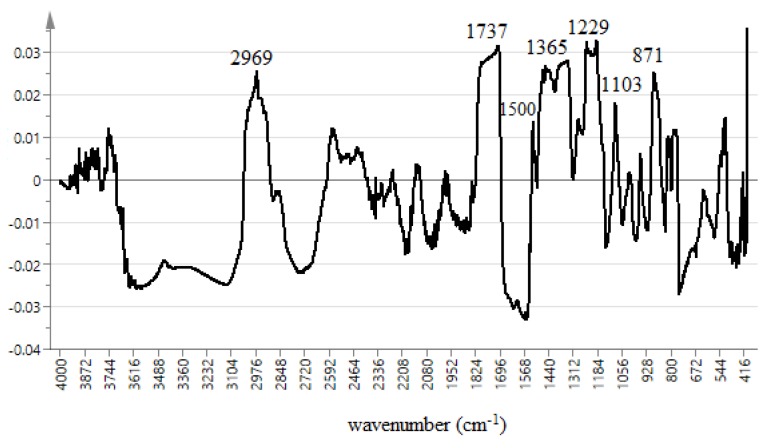
OPLS plot line loading of the extracts.

The advantage of the multivariate modal is to find the relationship among the complex data [33]. The data obtained from FT-IR spectrum allows identification of correlation of bioactivity and functional groups. OPLS model is suitable because it shows the relationship between FT-IR spectrum referred to as X and Y, the IC_50_ of α-glucosidase inhibitory activity (X = 3601, Y = 1). This model shows 4 components (1 + 3 + 0). OPLS score plot of salak extracts was shown in Figure 2. The 100%, 80%, and 60% samples extracts were clearly separated into negative semicircle while the other 40%, 20%, and 0% were clustered in the positive semicircle. The active extracts were strongly correlated with (C–H, C=O, C–N, N–H, C–O, and C=C) functional groups as shown in Figure 3.

### 2.5. Validation and Cross-Validation OPLS Model

**Figure 4:**
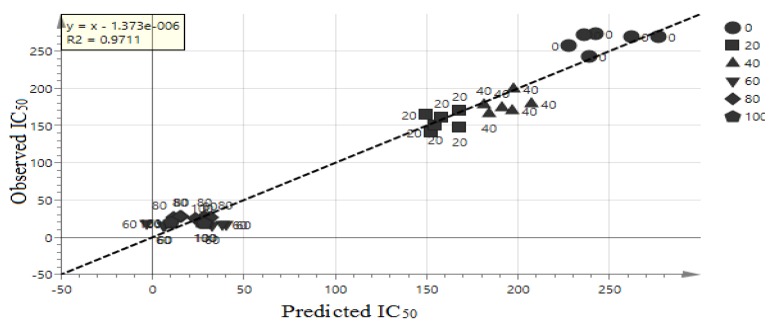
OPLS plot line loading of the extracts.

**Figure 5:**
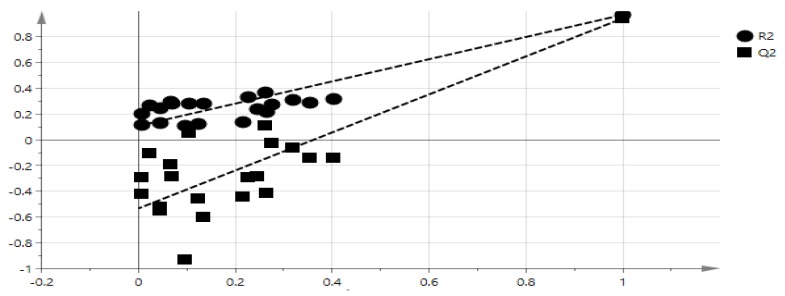
The OPLS model validation with 20 permutations.

The goodness of fit and the predictability of the OPLS model result can be validated by checking *R^2^* [33]. The goodness of plot predicted values is *R^2^* > 0.9 [34], and this model showed *R^2^* = 0.9711. Therefore, the model is considerably good as shown in Figure 4. The extrapolated Y value of the R^2^ and Q^2^ line was 0.109, −0.533 as shown in Figure 5. Generally, an intercept Y value were below 0.3, and −0.05 for R^2^ and Q^2^ respectively [30]. Therefore, this analysis showed that this model is valid. The external validation to validify the model was carried out using external salak fruit. A total of eight salak fruits were extracted using 60% concentration ethanol. Original and predicated values of α-glucosidase inhibitory activity of 60% ethanol extracts of the salak fruit were illustrated in Table 2. Hence, the model was also valid using external samples, therefore, this model can be used for rapid validation.

### 2.6. α-Glucoside Inhibiting Metabolites

**Figure 6:**
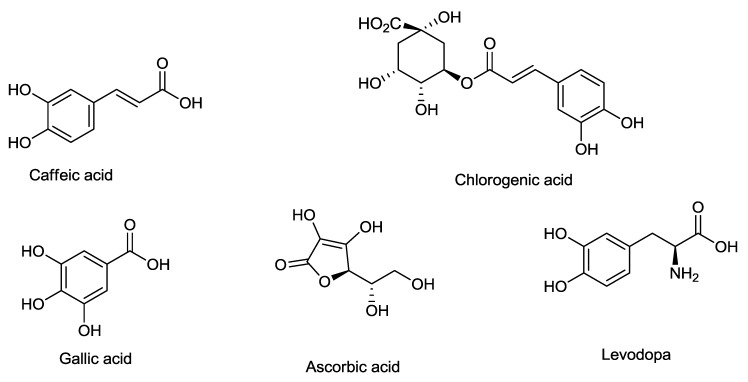
Chemical structure of different bioactive compounds from salak fruit reported to have α-glucosidase inhibitory activity.

The line loading plot from multivariate data analysis of different extracts of the salak fruit are shown in Figure 3. The plot provides information on the possible biological activities of the absorption peaks obtained from the FT-IR spectrum. It also assesses the characteristics of each spectrum that may promote or reduce α-glucosidase inhibition. Khatib et al. in 2017 adopted the use of this FT-IR fingerprinting as a means of correlating the functional groups with the antioxidant activity. It was concluded that functional groups such as C–N, C = O, C–O, C–H, and OH present in the extract influence the antioxidant activity. The uppermost loading plot represents the inhibitory activity, with a positive wave number at the uppermost loading plot representing inhibitory activity with a positive increase in enzyme inhibition. The peaks at 2969, 1737, 1500, 1365, 1229, 1103, and 871 cm^−1^ were observed to have positive values. These peaks represent the functional groups present in the constituents that are responsible for α-glucosidase inhibition. These are C–H, C=O, C–N, N–H, and C–O that are found in ester, amines, and aldehydes. The peaks at 2973 and 2719 cm^−1^ can be attributed to C–H stretching vibration of methylene and methyl groups. The peak at 1695 cm^−1^ depicts vibration of the ketonic group (C=O) present in aldehydes, esters, and fatty acids. The peak at 1439 is due to CH_2_ bonding vibration in esters and ethers, while that of 1311 cm^−1^ could be as a result of asymmetric stretching vibration in amines. The peak at 1100 may be due to C–O stretching of fatty acids and esters, S=O (SO_2_), and C–N (amines) ethers, and anhydride peaks at 850 and 543 cm^−1^ were a result of C–O stretching and deformation vibration in amines. Various phytochemical constituents were previously reported as an α-glucosidase inhibitor. Chlorogenic acid, caffeic acid, levodopa, and gallic acid were purified from salak fruit using temperature induced phase partitioning technique, while the ascorbic acid content determined through titration method. The outcome of this study shows compounds containing functional groups (C–H, C=C, C=O, and C–O) such as chlorogenic acid, caffeic acid, levodopa, and ascorbic acid which has been reported by previous researchers to be present in salak fruit [30,31] are responsible for α-glucosidase inhibition activity (Figure 6). Additionally, this result also shows that CHO, COOR, SO_2_, C–N, and N–H contain compounds that contribute significantly to α-glucosidase inhibition activity of salak fruit extracts. This outcome is being reported, for the first time, from this plant. We can, therefore, conclude that the presence of amines, fatty acids, esters, and sulfur-containing compounds may have a positive impact on α-glucosidase inhibition of salak fruit extracts. On the other hand, other peaks at the downmost post of the loading plot do not have a significant contribution to the inhibitory activity.

## 3. Materials and Methods

### 3.1. Chemicals

Glycine, *p*-nitrophenyl-α-d-glucopyronase (PNPG), and quercetin were supplied by Sigma-Aldrich^®^ (St. Louis, MO, USA). Ethanol (analytical grade) was purchased from R & M marketing (Essex, UK). Dimethyl sulfoxide (DMSO) and dipotassium phosphate were purchased from HmbG. α-Glucosidase enzyme was obtained from Megazyme (Wicklow, Ireland).

### 3.2. Samples and Extraction

Fresh salak fruits were randomly collected from a single location at Bukit Sagu plantation, Felda, Kuantan, Malaysia, on 15 May 2017. A sample was identified by a taxonomist at Kulliyyah of Pharmacy, International Islamic University of Malaysia, and deposited at an herbarium with the voucher specimen number of (PIIUM 0215). For cross-validation to predict their IC_50_ values, samples of salak fruits collected from eight different locations. The fruit was washed, peeled, and cut into 0.5 cm cubes. Then, it was dried using a freeze dryer. Finally, the dried flesh was grounded in a laboratory blender to a fine powder and kept at −80 °C. A total of 36 extracts were obtained using different ratios of ethanol–water as a solvent. Approximately 2 g of dried salak powder transferred to a 100 mL flask mixed with 40 mL of ethanol–water as a solvent in at different ratios (100%, 80%, 60%, 40%, 20%, and 0%) and ultrasonicated for 1 h. The mixtures were centrifuged for 20 min at 3500 rpm to obtain a clear supernatant solution. Later, it was filtered through a Whatman no 1 using Buchner funnel, and the solvent was evaporated using a rotary evaporator (Buchi^®^, Flawil, Switzerland) at 40 ± 1 °C. The crude extracts were stored and kept at –80 °C before further analysis [35]. The percentage yield of extracts using different ratios of ethanol–water were calculated:

$$\mathrm{Yield}\;\mathrm{of}\;\left(\%,w/w\right)=\frac{\mathrm{Weight}\;\mathrm{of}\;\mathrm{freeze}\;-\;\mathrm{dried}\;\mathrm{extract}\;\times 100\%}{\mathrm{Weight}\;\mathrm{of}\;\mathrm{dried}\;\mathrm{sample}}$$

### 3.3. α-Glucosidase Inhibitory Activity

The α-glucosidase inhibitory activity of the fruit was estimated according to Collins et al. [36] with slight modifications. A stock solution of the substrate *p*-nitrophenyl-α-d-galactopyranoside (PNPG) was prepared with 6 mg in 20 mL of 50 mM phosphate buffer pH 6.5. Quercetin (dissolved in DMSO) was used as a positive control. The salak extracts were dissolved in DMSO. A 100 μL of 30 mM phosphate buffer pH 6.5 added to each well, and 10 μL of different concentration (320, 160, 80, 40, 20, 10, and 5 μg/mL) of salak extracts were added to each well. Afterwards, 15 μL of α-glucosidase enzyme solution (0.02 U/well) was added, and allowed to incubate for 5 min at room temperature. After that, 75 μL of substrate (PNPG) was added to each well, and left for 15 min. By adding 50 μL of (2 M) glycine of pH 10, the reaction was stopped. The absorbance was measured using a spectrophotometer at 405 nm wavelength. The α-glucosidase inhibition was calculated based on the following equation:

α-glucosidase inhibitory activity (%) = [(A_control_ − A_sample_)/A_control_] × 100%

IC_50_ is the concentration of extract required to inhibit 50% of the α-glucosidase inhibitory activity.

### 3.4. Fourier Transform Infrared (FT-IR) Conditions

FT-IR spectrometer instrument (Perkin Elmer Inc., Massachusetts, USA) equipped with a horizontal attenuated total reflectance (ATR) device with diamond crystal was used in this experiment. The machine was allowed to equilibrate at room temperature (22 °C) before scanning. The FT-IR-ATR fingerprinting method developed by Sharif et al. (2014) [16] was used in this study. A small portion of each sample was placed neatly on the diamond crystal with a cleaned spatula. The FT-IR-ATR spectra of all samples were analyzed in the range between 400 and 4000 cm^−1^ at a resolution of 4 cm^−1^. The data was processed using Perkin Elmer Spectrum version 10.03.09 software (MA, USA). All spectrums were collected by using a rapid scan for each sample.

### 3.5. Statistical Analysis

Spectrums were converted into ASCII format after a baseline correction had been done. The converted ASCII data were pooled together with α-glucosidase inhibitory activity, and transferred into Microsoft Excel format before multivariate data analysis using SIMCA 14.0 (Umetrics, Umeå, Sweden). The orthogonal partial least square analysis was used in accordance with α-glucosidase inhibitory activity, and the functional groups obtained from FT-IR spectrum. The full FT-IR frequency regions of 400–4000 cm^−1^ were used at 4 cm^−1^ resolution. A total of 7189 variables attained. 36 independent observations were used to construct validation of the model. The validity of this model was tested by internal cross-validation based on the value R^2^Y and Q^2^Y cumulative. 

The results were analyzed using Minitab 17 (Minitab Inc., State College, PA, USA) by one-way analysis of variance (ANOVA) with Tukey’s comparison test. A 95% confidence interval applied, and the differences were considered significant at *p* < 0.05.

## 4. Conclusions 

In conclusion, the study has investigated the α-glucosidase inhibitory activity of salak fruit flesh extracts via in vitro technique. Conclusively, the results obtained showed that 60% hydroethanolic extract showed the highest activity, while the water extract exhibited the lowest. FT-IR fingerprint was successfully applied to a rapid investigation of α-glucosidase inhibition activity of salak fruit. A combination of FT-IR fingerprint with α-glucosidase managed to identify the functional groups susceptible to be involved for α-glucosidase inhibitory activity, such as (C–H, C=O, C–N, N–H, C–O, and C=C) compounds containing this fractional groups (chlorogenic acid, caffeic acid, levodopa, ascorbic acid, and gallic acid), which could be responsible for α-glucosidase enzyme inhibitor. 

## Figures and Tables

**Table 1:** Effect of solvent on yield and α-glucosidase inhibition activity of salak fruit.

Concentration of Ethanol–Water	Yield (%)	*α*-Glucosidase Inhibitory Activity IC_50_ (μg/mL)
0/100	69.4 ± 3.6 ^b,c^	271.46 ± 15.55 ^a^
20/80	67.2 ± 2.4 ^c^	156.12 ± 9.96 ^b^
40/60	67.7 ± 2.1 ^c^	175.81 ± 12.41 ^c^
60/40	72.6 ± 2.7 ^a,b^	15.94 ± 2.52 ^d,e^
80/20	75.5 ± 2.2 ^a^	26.82 ± 1.49 ^d^
100/0	20.2 ± 1.4 ^d^	19.15 ± 1.82 ^d,e^
Quercetin	ND	4.89 ± 0.48 ^e^

Values represented as mean ± SD of six replicates. Values represent with different superscripts are significantly different (*p* < 0.05) as measured by Tukey’s comparison test. ND = not determined

**Table 2:** Original and predicated α-glucosidase inhibitory activity of 60% ethanol extracts of salak fruit.

Sample	Actual α-Glucosidase Activity IC_50_ (μg/mL)	Predicted α-Glucosidase Activity IC_50_ (μg/mL)
1	33.11 ± 3.21 ^c^	Highly active
2	13.7 ± 1.18 ^d^	Highly active
3	54.44 ± 4.71 ^b^	Moderately active
4	120.33 ± 15.12 ^a^	Not active
5	45.45 ± 4.46 ^b,c^	Moderately active
6	50.66 ± 5.22 ^b^	Moderately active
7	14.71 ± 0.81 ^d^	Highly active
8	27.66 ± 2.40 ^c,d^	Highly active

The values are the means ± standard deviations *n* = 3, ^a,b,c,d^ Each different letter is significantly different (*p* < 0.05).
